# Lead poisoning among opium users in Iran: an emerging health hazard

**DOI:** 10.1186/s13011-017-0127-0

**Published:** 2017-10-05

**Authors:** Mohammad Mahdi Hayatbakhsh, Zohreh Oghabian, Elvira Conlon, Samaneh Nakhaee, Ali Reza Amirabadizadeh, Mohammad Javad Zahedi, Sodief Darvish Moghadam, Bighan Ahmadi, Somayeh Soroush, Jan Aaseth, Omid Mehrpour

**Affiliations:** 1 Department of Gastroenterology, Afzalipour Hospital, Kerman University of Medical Sciences, Kerman, Iran; 2 Gastroenterology and Hepatology Research Center, Kerman University of Medical Sciences, Kerman, Iran; 3 Institute of Basic and Clinical Physiology Sciences, Kerman University of Medical Sciences, Kerman, Iran; 4 Department of Pharmacology and Toxicology, Faculty of Pharmacy, Kerman University of Medical Sciences, Kerman, Iran; 5 Department of Clinical Toxicology, Afzalipour Hospital, Kerman University of Medical Sciences, Kerman, Iran; 6 Endocrinology and Metabolism Research Center, Kerman University of Medical Sciences, Kerman, Iran; 7 Public Health England, London, UK; 8 Cardiovascular Diseases Research Center, Birjand University of Medical Sciences, Birjand, Iran; 9 Medical Toxicology and Drug Abuse Research Center (MTDRC), Birjand University of Medical Sciences, Moallem Avenue, Birjand, 9713643138 Iran; 10 Inland Norway University of Applied Sciences, Elverum, and Research Department, Innlandet Hospital, Brumunddal, Norway

**Keywords:** Lead, Opium, Lead poisoning, Drug adulterants

## Abstract

**Background:**

Lead (Pb) poisoning among people using opium has been an increasing problem in Iran. The present study highlights the clinical effects of lead toxicity associated with opium use in Iran, Kerman province.

**Methods:**

Between January 2016 and June 2016, patients with signs and symptoms of Pb poisoning were questioned to assess whether they had a history of opium dependency. In total, 249 patients were enrolled onto this cross-sectional study, all were opium dependent. Para-clinical data including blood lead level (BLL), demographic information, user preferences, and symptoms were obtained.

**Results:**

The patients used either opium (83.9%), refined opium (6.4%) or a combination of both (9.7%) via ingestion (71.9%), smoking (8.4%) or a combination of both (19.7%). The overall median BLL was 80.0 μg/dL [IQR: 51.7–119.0]. The median BLL did not differ significantly between opium and refined opium users. Further, BLL was not significantly affected by the type of substance, route of use, duration of use, or daily quantity consumed. Common symptoms included abdominal pain (86.9%), constipation (75.8%), anorexia (71.5%) and nausea (54.7%). Linear regression analysis showed log of BLL was significantly associated with abdominal pain, myalgia and anorexia.

**Conclusions:**

The study unravelled an increase in opium-related Pb poisoning in the Kerman province. Raised awareness of this emerging Pb source and investigation of its aetiology is recommended. Pb poisoning should be considered among the primary differential diagnosis of opium users with gastrointestinal symptoms.

## Background

Historically, lead (Pb) has been one of the most widely used metals due to its useful properties such as low melting point, high density and ductility. Decades of widespread industrial and commercial use of leaded products, most notably petrol and paint, have left a legacy of environmental contamination which may contribute to chronic Pb exposure and acute Pb toxicity [1–4].

Chronic Pb poisoning appears progressively and is not specific [5]. Chronic exposure may trigger an acute presentation. The specific indications of acute lead toxicity involve abdominal pain (Pb colic), constipation, irascibility, neuropathy, muscle inflammation, joint pain, headache, anorexia, reduced sexual desire, concentration and short-term memory problems, nausea, lead lines on gums, anaemia, basophilic stippling of red blood cells, and kidney diseases [6–8]. Many outcomes of lead toxicity are related to its interactions with crucial components like calcium, enzymes and other proteins [9]. Lead poisoning can result from ingesting or inhaling lead compounds [10]. It can also occur as a result of dermal contact with organic lead, though to a lesser extent, or bone reabsorption of leaded bullets. Around 90–95% of absorbed lead is reserved in the cortical bone and teeth, liver, brain and kidneys. 75% of excreted lead is removed by the kidneys; the remainder is eliminated via the gastrointestinal tract, sweat, and skin or is accumulated in the nails and hair [7, 11, 12].

In adults, the greatest absorption of Pb occurs via the respiratory tract, where up to 95% can be absorbed in the lungs. When Pb is ingested approximately 5–20% is absorbed, largely in the duodenum.

Diagnosis of Pb poisoning is based on elevated Pb levels in the blood, specifically a BLL of ≥10 μg/dL. Discontinuation of contact with the lead source is a vital step in treatment. Most outcomes of lead poisoning can be reversed if lead toxicity is diagnosed swiftly and the source eliminated. Chelation therapy may be necessary in acute Pb poisoning in order to more rapidly reduce the BLL and facilitate recovery [6, 13].

Pb poisoning outbreaks around the world have typically occurred as a result of significant environmental contamination from activities such as Pb mining or smelting. Examples include the Pb poisoning outbreak in Zamfara State, northern Nigeria, in which several villages were affected by significant environmental Pb pollution due to mining activities. Examples of unusual Pb incidents are also available in the literature and include acute Pb poisoning occurring from the use of topical Ayurvedic treatment [14, 15] and culinary spices [16, 17].

There are several accounts of Pb toxicity arising from drug use, most involving opium [11, 20], though reports of cases involving marijuana [18] or methamphetamine [19] are also available. The earliest account of lead contaminated opium was in 1973 and was as a result of cross contamination from the kitchen equipment used to prepare opium for consumption [20].

The process of mass opium manufacture typically involves reducing opium poppies to brown sticky paste and sun drying, during which impurities such as Pb may be introduced or concentrated [21–23]. Refined opium, locally referred to as Shireh, is a strong opium produced by refining raw opium, known locally as Taryak, in boiling water, heating and filtering to remove insoluble materials [24].

Several sources of Pb contamination have been postulated in the literature: the adulteration of opium with Pb to increase weight, either at the point of production or at a local level [7, 25]; contamination from the processing equipment used during the production of the opium [20, 26]; and/or contamination of land on which opium is grown [27, 37]. It is suggested that other compounds like lead oxide, lead nitrate, lead acetate as well as Indian hair colours which contain lead can also be added to opium [27].

Some Iranian researches have analytically confirmed the presence of Pb in opium [6, 25]. Several others have reported lead toxicity in opium users [5, 26, 28, 29].

Iran shares a border with Afghanistan, a major producer of opium, and exportation of opium across this border has been identified as one of the main routes for transit of opium worldwide [30, 31]. Consequently, opium availability and use is a major issue in Iran, which may create a platform for opium adulteration and an increased risk of acute Pb exposure [30, 31].

It has been shown that Pb readily accumulates in the capsule of poppy plants grown in Pb contaminated soil [32]. Afghanistan, provides about 90% of the world’s opium [33]; the United Nations Office on Drugs and Crime reports that that average yield of opium has increased in certain parts of central and southern Afghanistan [34]. Afghanistan’s Pb mineral rich deposits and Pb mines are also largely distributed in these regions [35] so it follows that an increase in opium production grown on Pb contaminated land could potentially increase the Pb concentration in opium, this warrants further investigation.

It is feasible that multiple sources are responsible for the lead content in opium, herein lies a gap in the current knowledge. What is clear from published case reports and this current study, is that the number of opium users being admitted to hospitals for Pb poisoning appears to be increasing in Kerman and other parts of Iran [29, 36].

Meybodi determined that the prevalence of opium user patients with Pb poisoning in Tehran, Iran’s capital city, over a one-year period (2006 and December 2007) was limited to 25 patients [28]. In 2009 a study in Kerman showed no significant difference in the BLL of opium users (*n* = 50) and the control group (*n* = 43) [37].

Although in recent years there has been an increase in cases of lead poisoning due to opium use in some cities of Iran such as Birjand and Ardabil [8, 23], Pb poisoning related to opium use had until recently remained relatively rare and to our knowledge such poisoning was generally only reported as isolated cases.

The study herein highlights an acute increase in cases of lead poisoning of opium users reported over a short time period in Kerman Province, Iran. The aetiology of this increase is unknown. Given the severe outcomes of significantly increased serum level of Pb, this study also examines the clinical effects of acute Pb poisoning in opium users.

## Method

Between January 2016 and June 2016, all patients with signs and symptoms of Pb poisoning were questioned to ascertain whether they had a history of opium dependency. Opium dependency was evaluated according the Diagnostic and Statistical Manual of Mental Disorders - Fourth Edition﻿ (DMS-IV) criteria. All patients admitted to the hospital with suspected Pb poisoning were assigned to opium-dependant group (hereafter referred to as opium users) according to the DMS-IV criteria.

The assessment was conducted by a clinical toxicologist or gastroenterologist at Afzalipour hospital, the main toxicology and gastroenterology centre in Kerman province [38].

Two hundred forty-nine patients were enrolled onto this cross-sectional study using the census method. The inclusion criteria involved initial diagnosis of Pb toxicity, being over 18 years old, opium-dependency according to the DMS-IV criteria and having the desire to participate in the study. The exclusion criteria included those with pre-existing diseases (such as anaemia or liver, kidney and gastrointestinal conditions), those with a history of environmental or occupational exposure to lead as well as other drug use. According to the DSM-IV, an individual was classified opium-dependent when three of the following symptoms were experienced within the last 12 months: (1) tolerance to opium, (2) opium withdrawal when not consuming opium, (3) the need to increase the dose of consumed opium over time, (4) constant desire to quit or reduce opium use, (5) spending a great deal of time preparing to use, or recovering from the effects of use, (6) reducing time spent in social, occupational, or recreational environments, and (7) continuing opium consumption despite experiencing physical or psychological problems associated with its use.

Blood samples (5 ml) were collected from the cubital vein into heparinized tubes and stored at -20 °C. Blood Pb content was analysed by atomic absorption spectrometry (Varian Co., US) according to NIOSH standard method. A qualified doctor examined the patients and completed the questionnaire. Details of demographic data (age, gender and employment status), signs and symptoms, user preferences such as daily quantity consumed, route of administration and para-clinical data were collected according to a standardized checklist.

Biochemical concentrations of BLL, haematocrit, haemoglobin, blood urea, creatinine, alanine aminotransferase (ALT) and aspartate aminotransferase (AST) were recorded for subsequent analysis.

Ethical approval was granted by the Ethics Committee of Kerman University of Medical Sciences. Written and informed consent was taken from the patients after they were consulted on the study objectives, responsibilities of participants and researchers in the study, data collecting and recording techniques, confidentially and the use of data for publishing in scientific journals. No patient identifiable information is disclosed in this paper.

### Statistical analysis

Statistical analysis was performed using SPSS software (version 22.0, IBM Co., Chicago, IL). *P*-values <0.05 were considered as statistically significant. To explore the data, descriptive statistics (mean, standard deviation (SD), frequency, and percentage) were evaluated. The Shapiro-Wilk test was used to test normal distribution of numerical variables. The Student T-test or Mann-Whitney was used for two-group comparisons of continuous variables. Where the data followed a normal distribution, comparison was achieved by one-way ANOVA. Kruskal-Wallis test was used where data were skewed. In order to assess the relationship between BLL and haemoglobin, haematocrit, the Pearson correlation test was used. Associations between BLL and clinical symptoms, type of substance, route of use, duration of use or daily quantity consumed were evaluated by linear regression analysis; a level lower than 5% was considered significant.

## Results

In total, 249 patients were included in the study, 79% of which were male. The average age of cases was 48.5 ± 14.1 years (20–91 years). The mean BLL was 91.02 ± 59.83 μg/dL. The data were right-skewed, with a median BLL of 80.0 μg/dL [IQR: 51.71–119.00]. BLL ranged from 26 μg/dL to 350 μg/dL. According to Mann–Whitney U test, there was no difference in the median BLL of male (80.5 μg/dL [IQR: 51.73–120.0] and female (78.0 μg/dL [IQR: 43.0–113.75] patients (z = 0.83, *p* = 0.41).

The majority of patients (83.9% (*n* = 209) used solely raw opium, 6.4% (*n* = 16) used solely refined opium and 9.7% (*n* = 24) used a combination of both raw and refined opium. The median BLL of sole raw opium, refined opium and opium plus refined opium users was 80.50 μg/dL [IQR: 51.54–117.0], 110.0 μg/dL [IQR: 58.0–159] and 70.88[IQR: 39.0–91.0] respectively. According to Kruskal-Wallis test, there was no significant difference between BLL and type of substance (χ^2^ = 2.95, *p* = 0.23) (Table 1).

**Table 1:** Biochemical analysis of all users of raw opium, refined opium and combination users

Parameter	Type of substance				
	Opium *n* = 209	Refined opium *n* = 16	Opium + refined opium *n* = 24	F/*X* ^2^	*p*-value
BLL^a^ (μg/dL)	80.5 [51.5–117.0]	110.0 [58.0–159.0]	70.9 [39.0–91.0]	2.95	0.23
Haematocrit ^b^ (%)	33.5 ± 6.9	33.7 ± 3.7	34.4 ± 7.2	0.15	0.85
Haemoglobin ^b^ (g/dL)	10.5 ± 2.3	11.2 ± 2.1	10.6 ± 2.6	0.83	0.43
Blood Urea^b^ (mg/dL)	28.8 ± 13.2	26.1 ± 12.7	33.6 ± 21.7	1.19	0.72
Creatinine^b^ (mg/dL)	1.1 ± 0.9	0.9 ± 0.2	1.0 ± 0.1	0.10	0.91
AST^a^ (U/L)	46.0 [29.0–68.0]	27.0 [26.0–46.0]	41.5 [31.2–72.7]	2.86	0.23
ALT ^a^(U/L)	36.0 [20.0–60.0]	21.0 [14.0–33.0]	30.0 [19.0–45.0]	2.87	0.24

Blood lead level (BLL) median concentration [IQR]; hematocrit; hemoglobin; blood urea; creatinine; aspartate transaminase (AST) and alanine transaminase (ALT) mean concentrations (+SD) amongst the different user groups (raw opium; refined opium; and combination users)

Degrees of freedom for hematocrit, hemoglobin, blood urea, creatinine (df_b_ = 2, df_w_ = 246)

Degrees of freedom for BLL, AST, ALT (df = 2)

^a^ compared using Kruskal Wallis test

^b^ compared using one way ANOVA

One hundred seventy-nine patients (71.9%) were oral users, 21 (8.4%) smoked and 49 (19.7%) used a combination of smoking and ingesting. There was no significant difference in BLL associated with mode of use (Table 2). One-way ANOVA showed that there was no significant difference between duration of use (*F*_(2246)_ = 0.19, *p* = 0.83), daily quantity consumed (*F*_(2246)_ = 2.28, *p* = 0.11) and user drug preference (raw opium alone; refined opium alone; and combination of both) (Table 3).

**Table 2:** Median BLL across the users groups

Type of substance	Route of use	Blood lead level ^a^ (μg/dL)				
		Median [IQR]	Minimum	Maximum	*X* ^2^	*p*-value
Opium (taryak)	Smoking (*n* = 12)	80.0 [45.7–110.0]	36.0	126.0	0.17	0.92
	Oral route (*n* = 154)	80.0 [51.7–120.0]	26	350		
	Smoking + oral route (*n* = 43)	85.0 [51.0–116.0]	30	235		
Refined opium (shireh)	Smoking (*n* = 5)	96.5 [19.7–143.2]	70	150	2.37	0.31
	Oral route (*n* = 10)	140.0 [66.5–170.5]	58	250		
	Smoking + oral route (*n* = 1)	55.7 [55.7–55.7]	55.7	55.7		
Opium (Taryak) + refined opium	Smoking (*n* = 4)	48.3 [39.0–56.4]	39	58	2.51	0.28
	Oral route (*n* = 16)	72.1 [38.2–88.7]	27	200		
	Smoking + oral route (*n* = 4)	90.0 [54.8–156.5]	52	205		
Total	Smoking (*n* = 21)	70 [39.8–110.0]	36	150		
	Oral route (*n* = 180)	80.0 [51.2–120.7]	26	350		
	Smoking + oral route (*n* = 48)	85.0 [52.4–113.0]	30	235		

Median blood lead level (BLL) differences between routes of administration (smoking, oral and combination users) across the user groups (raw opium; refined opium; and combination users)

^a^ compared using Kruskal Wallis test

Degrees of freedom for BLL (df = 2)

**Table 3:** Duration of use and daily quantity of consumption against the type of substance

Type of substance	Duration of use ^a^ (years) Mean ± SD	Daily quantity of consumption ^a^ (g/day) Mean ± SD
Opium (*n* = 209)	15.25 ± 9.96	0.87 ± 0.54
Refined opium (*n* = 16)	16.84 ± 10.56	0.87 ± 0.75
Opium + refined opium (*n* = 24)	14.81 ± 8.27	1.22 ± 1.13
Test results	*F* = 0.19, *p* = 0.83	*F* = 2.28, *p* = 0.11

Mean (+SD) years of consumption and the quantity consumed in grams per day (g/day) across the user groups raw opium, refined opium and combination users

Degrees of freedom for time consuming and quantity consumed (df_b_ = 2, df_w_ = 246)

^a^ compared using one way ANOVA test

Similarly, there was no significant difference between duration of use (*F*_(2246)_ = 0.1, *p* = 0.90), daily quantity consumed (*F*_(2246)_ = 0.53, *p* = 0.59) and route of administration (smoking, oral, combination of both) according to one-way ANOVA (Table 4).

**Table 4:** Years of opium consumption and quantity consumed per day against the different routes of administration

Route of use	Duration of use (years) (mean ± SD)	Daily quantity of consumption (g/day) (mean ± SD)
Smoking (*n* = 21)	14.07 ± 9.18	1.16 ± 1.06
Oral route (*n* = 179)	15.13 ± 10.04	0.90 ± 0.57
Smoking + oral route (*n* = 49)	15.38 ± 8.83	0.93 ± 0.79
Test results	*F* = 0.1, *p* = 0.90	*F* = 0.53, *p* = 0.59

The mean (+SD) years of consumption and the quantity consumed in grams per day (g/day) across for the different routes of administration (smoking; oral; and combination users)

Degrees of freedom for time consuming and quantity consumed (df_b_ = 2, df_w_ = 246)

^a^ compared using one way ANOVA test

**Fig. 1:**
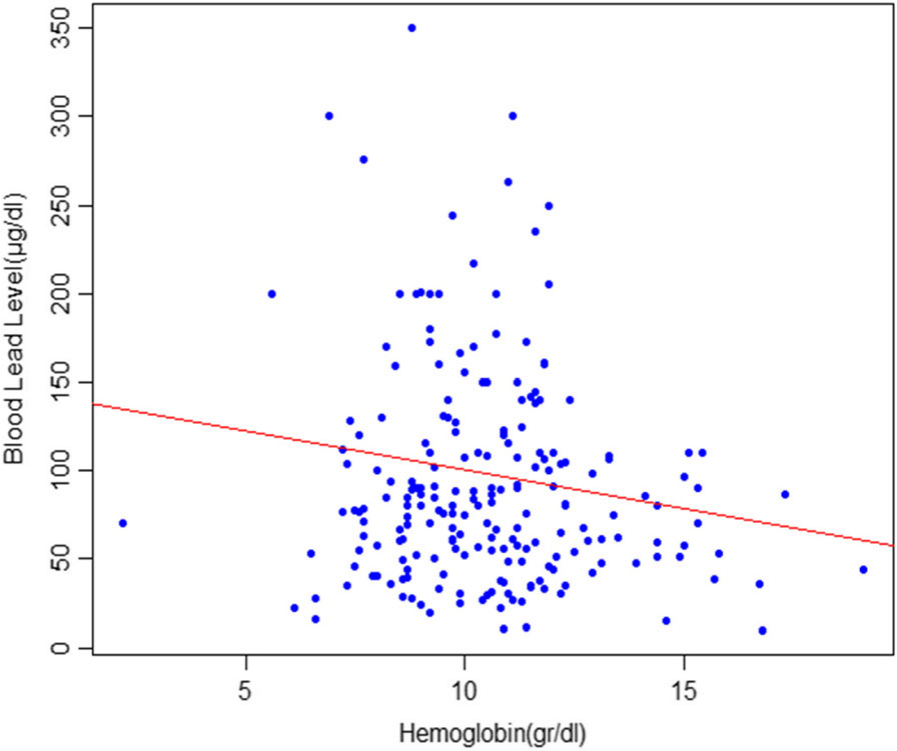
Relation between Blood Lead Level and hemoglobin

Table 1 shows the median BLL, haematocrit, haemoglobin, blood urea, creatinine, AST and ALT concentrations of the patients. Spearman’s r indicated an association between haemoglobin and BLL (*r* = −0.14, *p* = 0.04) among all user groups (Fig. 1). There was no significant relationship between BLL and haematocrit (*r* = 0.13, *p* = 0.06). Blood urea and creatinine levels were unremarkable among all groups. Compared to reference levels, 25% of the patients had elevated levels of ALT in the groups that used raw opium or raw opium plus refined opium. ALT was not elevated amongst the patients using solely refined opium. 42.4% of the patients had increased levels of AST in the groups that used raw opium or raw opium plus refined opium. There were no cases with elevated AST in the group using solely refined opium. There was a marked difference in AST in the male and female patients with 37% of the male patients exhibiting elevated AST compared to just 7.4% for female patients.

The six-month prevalence of signs and symptoms were as follows:

Gastrointestinal complains including abdominal pain (86.9%), constipation (75.8%), anorexia (71.5%) and nausea (54.7%) were the most common signs and symptoms. Other signs and symptoms included weakness (47.4%), arthralgia (38.1%), myalgia (33.2%), insomnia (32.8%) and peripheral neuropathy (3.7%).

BLL concentrations were right-skewed and were natural log-transformed for linear regression analysis. Geometric mean and ratio of geometric mean estimates were back-transformed. Our data indicated the linear regression model was statistically significant (*F* = 1.98, *p* = 0.04). Linear regression analyses for log of BLL were not significantly associated with type of substance (B = 0.36, *t* = 1.67, *p* = 0.09); route of use (B = 0.23, *t* = 1.44, *p* = 0.15); duration of use (B = 0.009, *t* = 0.63, *p* = 0.53) and daily quantity consumed (B = 0.08, *t* = 0.29, *p* = 0.77). Additionally, linear regression analysis showed log of BLL was significantly associated with abdominal pain (B = 0.98, *t* = 1.89, *p* = 0.03), myalgia (B = 1.16, *t* = 2.12, *p* = 0.006) and anorexia (B = 0.58, *t* = 1.79, *p* = 0.04) (Table 5).

**Table 5:** Univariate linear regression analysis for associations between BLL and clinical symptoms, type of substance, route of use, duration of use or daily quantity of consumption

Variable	B (SE)	95% CI	t	*p*-value
Type of substance (opium)	0.36 (0.22)	−0.1, 0.79	1.67	0.09
Route of use (oral route)	0.23 (0.16)	−0.1, 0.55	1.44	0.15
Duration of use	0.009 (0.01)	−0.02, 0.04	0.63	0.53
Daily quantity of consumption	0.08 (0.26)	−0.44, 0.60	0.29	0.77
Abdominal pain (yes)	0.98 (0.25)	0.85, 1.05	1.89	0.03
Constipation (yes)	0.13 (0.31)	−0.09, 0.68	0.75	0.68
Arthralgia (yes)	0.05 (0.33)	−0.05, 0.24	0.23	0.88
Myalgia (yes)	1.16 (0.34)	1.08, 1.38	2.12	0.006
Anorexia (yes)	0.58 (0.30)	0.34,0.72	1.79	0.04
Nausea (yes)	0.32 (0.33)	−0.08, 0.65	0.99	0.33
Peripheral neuropathy (yes)	0.71 (0.69)	−0.1, 0.98	1.12	0.30
Weakness (yes)	0.19 (0.33)	−0.05, 0.85	0.61	0.55
Insomnia (yes)	0.21 (0.28)	−0.04, 0.83	0.77	0.44

*F* = 1.98, df_b_ = 13, df_w_ = 236, *p*-value = 0.04

## Discussion

Pb poisoning in opioid users may currently be one of the most important forms of non-occupational Pb poisoning in Iran. Opium use is widespread in Iran and the clinical consequences of large plasma concentrations of Pb can severe and debilitating.

Although, in general, drug services for users of opium are extensive in Iran, a robust public health response to the growing problem of Pb contaminated opium appears to be lacking; the relatively small numbers previously affected may not have warranted great resources.

The overall median BLL 80.0 μg/dL [IQR: 51.7–119.0] (mean BLL 91.02 ± 59.83 μg/dL) of opium users in the current study was markedly elevated compared to other studies [6, 25, 39–41] which implies that the concentration of lead in opium may be increasing. For example, Khatibi-Moghadam et al., Ghaemi et al. and Salehi et al. reported a mean BLL in opium users of 7.14 ± 1.41 μg/dL (*n* = 40), 8.03 ± 6.03 μg/dL (*n* = 33) and 21.9 ± 13.2 μg/dL (*n* = 22) respectively [7, 23, 42]. Gastrointestinal symptoms, including abdominal pain, had the highest prevalence rate over the study period. Abdominal discomfort in these patients can be mistaken for many other diseases, and as a consequence may lead to unnecessary gastrointestinal imaging, laboratory tests, time wasting and increased risk of deteriorating condition [43]. For instance, Meybodi et al. and Mokhtarifar et al. presented examples of patients with abdominal pain caused by Pb poisoning which were mistakenly diagnosed as kidney stones, cholecystitis, acute abdomen or generalized ileus [28, 44]. As a result, it was suggested that an analysis of blood Pb should be included while evaluating patients who use opium and have unexplained abdominal pain [6].

The present study indicated a weak relationship between BLL and haemoglobin, Domeneh et al. and Beghé et al. also reported a similar weak relationship, however, in these two studies, age was reported as a predictor of anaemia. Poor nutrition and lack of adequate self-care can also accompany advanced drug use and may contribute to anaemia particularly in older drug users [29, 45]. In our study, the majority of participants were in their middle age.

Biomarkers for liver injury were compared across groups and to reference levels. There were elevated plasma levels of ALT and AST in 36% and 42.4% of cases respectively, though interestingly, not in those using the refined opium (Table 1). The reason for this is unclear as no other associations, including BLL and liver disease, could be established. Is it feasible that other metals and contaminants were present in greater concentrations in the raw opium but due to scope and design of the study these factors were not investigated. It would be valuable to include analysis of other known opium contaminants in future study designs. There were also stark differences in levels of AST in males and females; females had far less incidence of elevated AST. This also appears to be unrelated to BLL as there were no differences in BLL between the males and females. There were no appreciable differences in renal biomarkers.

Most individuals in this study were oral consumers of opium, similar results have been reported in other studies [46, 47]. It was expected that smokers of opium would have increased BLLs in comparison to those that orally consumed, however, there was no significant difference among these groups. Complete volatilization of Pb is dependent on the temperature exerted upon it; it is possible that the temperature generated in the smoking of opium does not fully liberate Pb vapour or a portion of the vapours escape without being inhaled resulting in decreased absorption via inhalation. Absorption following ingestion of opium may be affected by nutritional status, pH and transit through the digestive tract. Opioids decrease gastrointestinal motility; thus, constipation may result in greater absorption of Pb into the bloodstream through prolonged intestinal exposure. Future studies could include a comparison of the level of constipation against BLL. It would also be of interest to trace a possible “cocktail effect”, i.e. to explore the possible combined toxic actions of other narcotics and Pb and/or other heavy metals, that may lead to lower critical levels for Pb exposure in drug users compared to healthy population.

The frequency of raw opium users (*n* = 209) was significantly greater than refined opium users (*n* = 16), which may simply be due to differences in the popularity of raw opium over refined opium, potentially due to cost or drug effect. It could be speculated that a reduction in opium Pb content due to the refinement process decreases the incidence of Pb poisoning in users of refined opium. However, in our groups, the BLL was not significantly different between refined opium and raw opium users. Further, the refinement process, which involves boiling raw opium pulp, is unlikely to significantly remove heavy metals.

The difference in group sizes places limitations on the interpretation of our findings. It would be of interest to conduct a study with comparable group sizes, ideally incorporating controls consisting of participants who use opium without Pb poisoning and participants not using opium but with Pb poisoning. Another limitation of this study was that no analysis of opium Pb content was conducted because of difficulties in obtaining opium samples from the patients; this should be considered for future studies. Although not confirmed by opium analysis, patients’ drug use, diet, patient history, lifestyle, occupation, and the presence of pica (the consumption of non-nutritive substances) were examined and no associations were found. During the study, there were no cases of lead poisoning admitted to the hospital in populations not using opium; it is therefore reasonable to assume that the source of lead poisoning in the study group was the opium.

## Conclusion

Based on the findings of our study it is apparent that there has been an increase in the frequency of acute Pb poisoning amongst opium users in the Kerman province. The reason for this spike in numbers remains unclear and warrants investigation. Raised awareness of this Pb source is recommended. Pb poisoning should be considered among the primary differential diagnoses of opium users with gastrointestinal symptoms to assist in early diagnosis and prevent further complications.

## Abbreviations

ALT: Alanine aminotransferase
AST: Aspartate aminotransferase
BLL: Blood lead level
IQR: Inter quartile range
Pb: Lead
